# Supplementary material for: Accurate detection of m6A RNA modifications in native RNA sequences
Source: Nat Commun. 2019 Sep 9;10:4079. doi: 10.1038/s41467-019-11713-9 (PMC6734003; doi:10.1038/s41467-019-11713-9)
Supplement: Supplementary file 1 — Supplementary Information New [file 41467_2019_11713_MOESM1_ESM.docx]

**SUPPLEMENTARY INFORMATION**

Accurate detection of m6A RNA modifications

in native RNA sequences

Huanle Liu^1,2,$^, Oguzhan Begik^1,2,3,$^, Morghan C Lucas^1,4^, Jose Miguel Ramirez^1^, Christopher E. Mason^5,6,7^, David Wiener^8^, Schraga Schwartz^8^, John S. Mattick^2,3,#^, Martin A. Smith^3,9^ and Eva Maria Novoa^1,2,3,4*^

*^1^Centre for Genomic Regulation (CRG), The Barcelona Institute of Science and Technology, Dr. Aiguader 88, Barcelona 08003, Spain*

*^2^Department of Neuroscience, Garvan Institute of Medical Research, Darlinghurst, NSW, 2010, Australia*

*^3^St-Vincent’s Clinical School, UNSW Sydney, Darlinghurst, NSW, 2010, Australia*

*^4^Universitat Pompeu Fabra (UPF), Barcelona, Spain*

*^5^Department of Physiology and Biophysics, Weill Cornell Medicine, New York 10021, NY, USA*

*^6^The Feil Family Brain and Mind Institute, Weill Cornell Medicine, New York 10021, NY, USA*

*^7^The WorldQuant Initiative for Quantitative Prediction, Weill Cornell Medicine, New York 10021, NY, USA*

*^8^Department of Molecular Genetics, Weizmann Institute of Science, Rehovot, Israel*

*^9^Kinghorn Centre for Clinical Genomics, Garvan Institute of Medical Research, Darlinghurst 2010, NSW, Australia*

*^$^These authors contributed equally*

*^#^Present address: Green templeton College, Oxford OX2 6HG, United Kingdom*

* Correspondence to: Eva Maria Novoa (eva.novoa@crg.eu)

**SUPPLEMENTARY FIGURES**

**Supplementary Figure 1**. Replicability of the features extracted, when comparing across replicates (**A**) Comparison of features between m6A-modified datasets (replicate 1 and replicate 2) and m^6^A-unmodifed datasets (replicate 1 and replicate 2). Each dot corresponds to a different nucleotide of the synthetic constructs (n=9978) (**B**) Comparison of features and across datasets, comparing m6A-modified and unmodified datasets of replicate 1 (upper panels) and of replicate 2 (lower panels).

**Supplementary Figure 2**. Replicability of the base-called features of GGACU k-mers, for each position of the k-mers. Base-called features of m^6^A-modified datasets are depicted in red, whereas those from unmodified datasets are depicted in blue. Error bars indicate s.d.

**Supplementary Figure 3**. ROC curves of SVM trained with single features compared to combined features. Performance of each replicate is shown separately in each plot.

**Supplementary Figure 4**. Replicability of the direct RNA sequencing experiments across biological replicates, expressed as log counts for each gene. Each dot represents a gene, and the *ime4*gene has been highlighted in red. Correlation values shown correspond to Spearman’s rho.

**Supplementary Figure 5**. Base-called features (base quality, insertion frequency and deletion frequency) of RRACH 5-mers known to contain m^6^A modifications (left panels) compared to those that are not known to contain m^6^A modifications (right panels). Only features corresponding to the modified nucleotide (position 0) are shown. Features extracted from *wt* yeast reads (m^6^A-modified) are shown in blue, whereas those from *ime4*∆ (unmodified) are shown in red. Error bars indicate s.d.

**Supplementary Figure 6**. SVM performance is dependent on per-site read coverage. (**A**) ROC curves depicting the effect of per-site read coverage in SVM performance (number of reads per-site tested: 1, 2, 3, 5, 10, 20, 30, 50, 100, 200 or 500). Different read coverages have been simulated by random subsampling of reads. For each simulated dataset, area under the curve (AUC) and the accuracy are shown. (**B**) Per-site read coverage in yeast wild-type (WT) and ime4∆ knock-out (KO) strains, for each biological replicate. The median coverage for all sites is depicted in red.

**Supplementary Figure 7.**  Comparison of base-called features at position 0 in two different RRACH k-mers (GGACA and GGACC). Base-called features from ime4∆ (red) and wild-type (blue) strains are shown, both in the form of boxplots and density histograms. Only known m^6^A-modified RRACH sites in *S. cerevisiae* have been included in these plots. Error bars indicate s.d.

**Supplementary Figure 8.** TapeStation output of the quality and quantity of the m^6^A-modified and unmodified *in vitro* transcribed products

**Supplementary Figure 9**. Comparison of base-called features using different base-calling algorithms: Albacore 2.1.7, Albacore 2.3.4 and Guppy 2.3.1. (**A, B, C**) Per-base quality scores, mismatch frequencies and deletion frequencies at position 0, comparing m^6^A-modified reads (blue) to unmodified reads (red), using either Albacore version 2.1.7 (A), Albacore version 2.3.4 (B) or Guppy 2.3.1 (C). (**D, E**) Comparison of mismatch frequencies at position 0, grouped by the reference nucleotide, both for unmodified datasets (D) and m^6^A-modified datasets (E). Mismatch frequencies at A positions are consistently increased in m^6^A-modified datasets, and for all three base-callers tested. Error bars indicate s.d.

**SUPPLEMENTARY TABLES**

**Supplementary Table 1.** Sequencing output and reads mapping metrics of the ‘curlcake2 datasets

|  | **Unmodified** | | **m^6^A-modified** | |
| --- | --- | --- | --- | --- |
| **Sample Type** | Rep1 | Rep2 | Rep1 | Rep2 |
| **No. of sequenced reads** | 66,736 | 846,595 | 134,374 | 638,860 |
| **No. of base-called reads** | 63,047 | 745,472 | 130,998 | 535,069 |
| **N50** | 1457 | 1981 | 1314 | 1596 |
| **Mean Quality** | 6.9 | 6.8 | 5.8 | 5.2 |
| **Total sequencing  throughput (Gb)** | 0.06 | 0.801 | 0.122 | 0.574 |
| **No. of base-called reads** | 63,047 | 745,472 | 130,998 | 535,069 |
| **No. of mapped reads** | 47, 341 | 603,054 | 11,839 | 93,434 |

**Supplementary Table 2.** Accuracy of EpiNano predictions in m6A motifs (RRACH), relative to control motifs (RR[CGT]CH), using different feature combinations, and for both replicates.

|  |  | m6A motif | | Control | |
| --- | --- | --- | --- | --- | --- |
|  |  | REP1 | REP2 | REP1 | REP2 |
| 1 feature | base quality (q) | 86.60 | 85.57 | 61.92 | 57.49 |
|  | mismatch_frequency (mis) | 73.71 | 72.68 | 50.09 | 50.65 |
|  | insertion frequency (ins) | 50.03 | 50.20 | 50.09 | 50.09 |
|  | deletion frequency (del) | 70.62 | 70.10 | 50.09 | 50.09 |
|  | Current intensity (curr) | 53.61 | 55.67 | 50.20 | 52.87 |
|  | Current stdev (stcurr) | 63.92 | 65.98 | 48.98 | 51.02 |
|  |  |  |  |  |  |
| 2 features | q+mis | 89.18 | 88.14 | 61.55 | 57.49 |
|  | q+del | 88.14 | 88.14 | 62.48 | 57.49 |
|  | q+Current | 85.05 | 86.08 | 67.28 | 58.60 |
|  | q+StCurrent | 89.18 | 87.11 | 62.66 | 54.34 |
|  |  |  |  |  |  |
| 3 features | q+mis+del | 90.72 | 88.66 | 60.63 | 59.89 |
|  | q+mis+curr | 86.60 | 88.14 | 69.87 | 59.15 |
|  | q+mis+stcurr | 89.69 | 88.14 | 66.36 | 59.15 |
|  |  |  |  |  |  |
| 4 features | q+mis+del+curr | 90.72 | 90.72 | 66.36 | 58.41 |
|  | q+mis+del+stcurr | 90.21 | 89.18 | 60.63 | 54.71 |
|  |  |  |  |  |  |
| 5 features | q+mis+del+curr+stcurr | 88.14 | 88.66 | 67.47 | 57.67 |

**Supplementary Table 3.** Sequencing statistics of *S. cerevisiae* polyA+ selected RNAs direct RNA sequencing runs, sequenced in biological triplicates.

| **Samples** | **No. sequenced  reads** | **No. basecalled  reads** | **No. mapped  reads** | **% basecalled** | **% mapped** | **No basecalled and re-squiggled reads** | **% basecalled and resquiggled** | **% lost due to re-squiggling** |
| --- | --- | --- | --- | --- | --- | --- | --- | --- |
| KO1 (ime4∆) | 695,082 | 536,225 | 322,122 | 77.15 | 60.07 | 286,486 | 41.22 | 35.93 |
| WT1 | 1,011,064 | 926,729 | 522,012 | 91.66 | 56.33 | 280,651 | 27.76 | 63.90 |
| KO2 (ime4∆) | 577,856 | 474,694 | 240,077 | 82.15 | 50.58 | 210,485 | 36.43 | 45.72 |
| WT2 | 632,529 | 526,035 | 249,694 | 83.16 | 47.47 | 236,652 | 37.41 | 45.75 |
| KO3 (ime4∆) | 943,961 | 861,001 | 466,702 | 91.21 | 54.20 | 360,600 | 38.20 | 53.01 |
| WT3 | 944,079 | 801,145 | 407,858 | 84.86 | 50.91 | 349,746 | 37.05 | 47.81 |
|  |  |  |  |  |  |  |  |  |
| SUM (ime4∆) | 2,216,899 | 1,871,920 | 1,028,901 | 84.44 | 54.97 | 857,571 | 38.68 | 45.76 |
| SUM (WT) | 2,587,672 | 2,253,909 | 1,179,564 | 87.10 | 52.33 | 867,049 | 33.51 | 53.59 |
| TOTAL | 4,804,571 | 4,125,829 | 2,208,465 | 85.87 | 53.53 | 1,724,620 | 35.90 | 49.98 |

**Supplementary Table 4**. Comparative algorithm performance for the prediction of m6A modification in yeast samples (wt and ime4∆), using sites identified by Illumina as ‘true positive’ set.

|  | **EPINANO v.1.0** | **TOMBO**  **v.1.5** |
| --- | --- | --- |
| **TP** | 117 | 220 |
| **FN** | 252 | 149 |
| **FP** | 7,198 | 18,370 |
| **TN** | 53,596 | 42,424 |
| **Total sites analyzed** | 61,163 | 61,163 |
| **Accuracy** | 0.88 | 0.70 |
| **Sensitivity (TPR)** | 0.32 | 0.60 |
| **Specificity (1-FPR)** | 0.88 | 0.70 |
| **Area Under Curve (AUC)** | 0.68 | 0.64 |

**Supplementary Table 5.** Quality and yield of the *in vitro* transcribed ‘curlcake’ RNAs sequenced

| IVT product | Replicate | Volume (ul) | Qubit Read (ng/ul) | 260/280 | 260/230 |
| --- | --- | --- | --- | --- | --- |
| Curlcake1 Unmodified | 1 | 10 | 520 | 2.52 | 3 |
| Curlcake 2 Unmodified | 1 | 10 | 467 | 2.56 | 3.11 |
| Curlcake 3 Unmodified | 1 | 10 | 335 | 2.66 | 3.4 |
| Curlcake 4 Unmodified | 1 | 10 | 590 | 2.49 | 2.72 |
| Curlcake1 m6A-modified | 1 | 10 | 429 | 1.82 | 2.8 |
| Curlcake 2 m6A-modified | 1 | 10 | 550 | 1.91 | 2.76 |
| Curlcake 3 m6A-modified | 1 | 10 | 580 | 1.84 | 2.8 |
| Curlcake 4 m6A-modified | 1 | 10 | 310 | 1.9 | 2.89 |
| Curlcake1 Unmodified | 2 | 10 | 372 | 2.1 | 2.79 |
| Curlcake 2 Unmodified | 2 | 10 | 328 | 2.09 | 2.31 |
| Curlcake 3 Unmodified | 2 | 10 | 302 | 2.17 | 2.49 |
| Curlcake 4 Unmodified | 2 | 10 | 304 | 2.13 | 2.46 |
| Curlcake1 m6A-modified | 2 | 10 | 204 | 1.8 | 2.62 |
| Curlcake 2 m6A-modified | 2 | 10 | 242 | 1.86 | 2.49 |
| Curlcake 3 m6A-modified | 2 | 10 | 200 | 1.85 | 2.48 |
| Curlcake 4 m6A-modified | 2 | 10 | 200 | 1.85 | 2.48 |

**SUPPLEMENTARY FILES**

**Supplementary Note 1.** Fasta sequences of the ‘curlcake’ constructs used in this work

Fasta sequences of Curlcakes

>cc6m_2244_t7_ecorv (Curlcake 1)

ATCATAATTAATACGACTCACTATAGGGAGATCCGGCTGTGGAAGTCGATGAATCAAAAACCTTGGACCCTATCTCACAACGAACGAGTAATCCTGCGTGGGACTACAAAGTCGTTTTCGTGGACCAGGCCTCGACCTCCCAAGCATATGGGGACGTCGCTCAAATCCACAGTAGACTATCTATTAACTAATTGTGGATCGATGCTGACGCTACCCAGGTCTAGTTCGAGCCAATAATGTGCTACGTCGCGAAAGCCAAACAACGCTAACACCCTATCGTTCATTTGATACGTAACATCAATCACCTTGCAACTAGCTCGAACGGTATGGTTCAACGAGGGTGTTAGGCAGCGAACAACCCAGAGCGTTTATCATACCATATGCTCAACATACTTCCAGAGAGTCTGTTGGTCTAACAAGGTATAGCGGCGAGCAACGGAAAAGATAGTGCGTAATAGTTCGAGGAGCACCGCATCCCCCTCGCCGTTTTCGGGCCGTTAATATGGGCCGTAACACACCGGAGTTTCATAAAACGGCCTTTCCGTTGCTCGGGTGAGGGCATACGTGCTGCCTGGAGGGTATCCTACATGGTTAAATGCTGCGCCCCGTTAGGAAGCAGCAAATAAGAGTCTACCCGGTCTACCGGTACAAAGTTCGTGAGAGTTCTTCATGCTCGTCTCATAAAGGACTGGCAAAGGCCTTTGTTGCGTCAATGAGGAAGCCAAGCGGCGAGGTCAAGGCCACGAGTACATGGGCCATTCTCCAGTATAGTGAAATAAGCCCACGCGGTAAACCAAATGTGGAGCACACTCCACGTTCCTGTTCGTGGGCCTATTGGGATATGTCTTTATCAGACAACCTTATTTACGCGTATACATGCGGCAGTAGGCCTCGCGGTCCTAAGGGTCAGGTGTTACCACTTACCTCAATTTTCTCTGGAGTAGTACTTAGACTACCATCTTTGTTGAGAAATCATCTCACCTCTCTAGTCAGGGATAGCGTCCACTGTCTAATTATACAACTATTCGATCAAGGTCGCAAGTGGATACTGAAAATCACTATGTAGAGCTTCGGAAAGCCTGAATTTTAAGTTATATCCTAAGAGCAGTGAACATTTCCAGCGATTTCCGTAGTTGCAATGTCAAACCCCGATCTAGGGTTAGAGTGCCTAGGTGCTATCCACGTGGTGTCCCTGGGAGGCTACCCTTCGTGGACATAACTCGGCAGACACTAGTCCGATCGCCAGGACAAAAATCAGCGACGAACCGGTCGAATCTTAAGCGAAATAGACGAGAAGTTTAGGCATGTTAAAGCGGGTGCCCGGCACTGGCGCGAGTAACGTTCCCTCCTCCATATCTATACACCAACCCCGAACCAGGAAGTGGGACTGGAGAGGTTTGGGTATCTTCAAAATTGTGCATTACTGTTGGAAAGGTTGGATGCTTCGCGCAATATCGATACCGATCTACTTGCGGTGAGCGCAAATTCGCGTAGCAAATAGTGACCCATCGCGGCCATCAATGCTGACAGGGAGCACAACCAGATCACGATAACTTGTAAAGGGGGTATAGCAGCTCACCGGAGGGAAATACTGCAATCATGGAGTCGTAAAACAGGGCCATGTCAACATCGTACGCGTTCGTCATTCTAGGTATTTATTCAAGGGGATAGAAGCGTGGAGTTATTGAATCCAAACCCAAGCCGCTGCAGCGAGGGCTACCACCCACGAGATCGGATGGTAGGGGGCGCCCGTGGGTAGGGGCCCTGGGTTATCACAAAAAGATCGCACTCTTCCTGGGCTTATGCGATACATCACCGGGAAATGATGCCACGTACCAGGATCGTTACGGGACCACGTCGAAGGCGCAGCAGCGAAGTGTGCTCTAGTATGTTTGTGAGTAGCACGGGTCGACGTTTATGCGTTCTCTTTCCTCCTAGATGGCAGCCTCCCCAATGATGGGAAGGACTTTTATCGACATGTGAACCGGCCCTAACGAGACAGGTCTTGGTCTTGGTATCGAGCAAGTATAACCAATTTCTTCACTCGCAGAAAAATGTTGACGAGGAGCAATTCTCTATTAACGCACGGTCTGCTATTTCGGGCCGGAGTCAAAGCTCGTATATCGTGACCTCTTGGGGATCGTGTCCCCGCTTGTGAATGTAGGCTCGCGGCCAACCCTCGTTACTATACGACGGGAGTGGGGAGTACGACATCAATAAAATAAGCAAAACAGCTCAAGATGCGACCTAGCCAATGATTTTAGGCGATCATTGGATCCgcgccgcgggccgcTCAATCTCAATCgcggcccgcggcgcGGAT

>cc6m_2459_t7_ecorv (Curlcake 2)

ATCATAATTAATACGACTCACTATAGGGAGACAGAAGCGGATTATGGGGGCGCCATTATCGCGCGCGTGCTACCCTGAATGATACAGAGCGTGTGTCCACGTCGAGCTGTTGCGTAAGTCGTCAGCATACGTCCCTTGGACCGTGCATATTCCTCAGTCACTACTTTCTAGACTCAATATCTTTCGATGCTCCCGTCGATCAAGTAACTCGCTGTCTCGCTCTGTGAGAGCAATAGCGCCTCGAGCTAAATGCCGACGCAAGGGGAATCATAGGAAAAGCCAGCAAAGCCCCTAAAAAAACGAGCATGGCTGTCATCGCTTGGGAGATTCGGGACTTAACAACGTCAGGCTAAGGGTAAAGGTTGGCCGTGTTAACCAGTTGCTCGGTCATGTCTCCGCTACCTGTGATCATATCGAAAACATAATAGCTAACTTGCCCGCAGATGGGCCGTGTGAAGCTGGTAGCCGACCCCACGGCCTTACCGGACACGCCCGGACCCCCGACACTCGTAGTAAAATTGATACATCGCGTTAATCGTGGGGCTGGCGCGTGCGACGGTTCTTGACATGTGTGGGTTATGTAAGTATAACAACCCTTGCGGCGTTGGCACGGCCCTAAGCTGAGAAAAAAGGTGCTCATAACTCAAACTTCGACGTTCCCCGTGGGTGGCGATAGGCATTGGTATGGTTTGGTCTTCTGCAAGTCGCCCAGGATGTGATCAATGTAACGAAAAGGAATGATAGTAAAAAGATCCGGCTGTGGAAGTCGATGAATCAAAAATCTTGGACCCTATCTCACAACGAACGAGTAATCCTGCGTGGGACTACAAAGTCATTTTCGTGGACGAGCCCTCGACCTCCCAAGCATATGGGGACGTCGCTCAAACCCACAGTAGACTATCAACTAACTATTGTGGCTCGAAGCTGACGCTACCCAAATCAGGTTCGAGCAAATAATGTGCTACGTCTCGAGAAACAACCCGAACACCCTGTCGTTCATTGGATACGTAACATCAATCACCTTGCAACTGCTCGCGGTATGGTTCAACGTGGTTGTTAGGCAGCGAACCACCCGGAGCGTTTATCACGCCATATCCTTAACAAGCCTCCAGAGAGTCTGTTGGTCTAACACTGTATAGCGGCGAGCGACGGAAAAGGTAGTGCGTAAAAGTGGATTAGCACCGCATCCCCCTCTCCGTTTTCTGGCCGTTAATATGTGTCGGAACAGACCGGATTTCATCACACGGCCTTTCCGTGCTGGGGTGAGGGCATACGAGATGCCTGGAGGGTATACTACTTGGGTAAATGCTGCGACCCGTAGGAAGCGGAATAAAATGTCCCCGGTCTCATGTACAATGTTCGCGCGAGTGCTCTATGCTCAGCTCATCCGTCACTGACTAAAGCCATTGCTGAGTCCATGAGGTAATAACGCGGCTCCGACAAGTCCCTAATAGTGTGCGCTTCTCCTGAATTGTCAAATGAGCCGACGATGTATTCCAAAGGCGGAGGCCACTCCACGTTCCTGTACGTTGGGGGATTGGGATATGCATTTCTTAGACCTGCTATTATACGCGTATAAACGCCCGTCGAGGTCGCGTTACTAATGGTGAAGTGATACCACACACCTAACGTCCAGCAGTATTTAGATACAAGATATTTGAGAACTCATTAACTCCTCTGGTCAGGGATGCTTCCACTGGACTCTAAGACTGTTTGATCAAGGTGGCCTATAGTTACGAATAGCTCTGTAAAGAGCTTCGGATGGCCAGATTTATTAGGTACACCATAGGACCAGTGAACATATACCGCGACATCGGTAAGTGTAATGCCACGATCTAGGGTGTGAGCGCCTAGGTGCTTTCACCAGGTCTGGGAGAGCCTGCCCTTCGTAGTCAGAACCCTCAGACTTAGTCCGATCGCCAAGTAAACATGACGTAGCCCCGCCGAAACGTTATATAGGCTAGAAGTATATGAAGGTTAAAGGGTTCCCGGCACTGCCGAGTCAATTCCTCCTATTTTTCAAGAGGCTGAAGACAGGAACCTGGTTTGTAGATGGTCGACTAGCTTTTAAACTGAACTACCAGCTAGGAGGTTTAGCGTACGATAAATTTCGATTCCGGTTTCTCAAGTTGAGCTCCCCTGACAGTGGTGCCTCGGGACACAATACGGCAATCTGTGATTACAGGGTCCAACACAGCAACCAGACATGCTAGTAAGATTAAATATATCAGAGCGGGGGGCACGCAGCCAGCCTTCCCCAGTAACGACGTTTCAGGCCCACTTCGCTTTAGTACGGAGTCGTCGTACTTCTGAAAATAGAGTAGGCGAATCGAAATCCCACCAAGACGTGCCCGGGGCTACAGCCCAGAAATGGGTGGGGCGGCCCGACTTGAGGCACATGTTATCGCTATGAGTACCGAAGATGAGAGGATGACATACTTAATTGAGTGGGTTGACGGGCCATGGTAGGTCATAGACAAATTCTAATTGGATCCgcgccgcgggccgcTCCATCTCCTACCgcggcccgcggcgcGGAT

>cc6m_2595_t7_ecorv (Curlcake 3)

ATCATAATTAATACGACTCACTATAGGGAGACGCAGAGTTAGTGGCGCTGGCATGTGGTAACCAAAACGGTCGTGATGATGCGTTCTCTTCCGAATTTGTCCTCATACAGAGGGAGCTAACCTTATTTCCAGTCTTAAGCGAGACTCGCAAGAAGGCATCGTTGCACACTAGATTGATTCAATGCAAAAGCAGACGAAAGGATAAGAGTGTCTTTCTATATTGCATCATGCCGTCTTCGAATGATCGGCTAATCTACACATAGCCGGCCGCGCCAGTTGTCTCTCTGCCAAACGATTATGTAGGGCTCTCGGCAGGATCACTTATCTGAGACGCGAAGTTCCATAATTTTAGGATTCGACGCAATTGGTACGCACGGGGTTTTGAATGTAACTGTCCGGACTGGTGGAGTGAGTTGCGTCAGTCGCACAAGGGCGCGGACCTTTTTGTTTCCTTTAACGGCGTCTACTATGGCGTGACCCCTTAGCCTAAACCATGCGCCCATGTCAGCGCTCTTGTGTGGGCAACGGACGCCGCCTCTACGCCTTACAACAGTTCAGCCGTACATAACAGCTGGAACGGGTACCTATGTTTAAGTAGTAGCTATCCAGGACTTTCGCCTGTGCAAGCGCATTGAAACTTGTCGAACTTCAGATAACCCCGTGGCAGTGCCGGGAACTGGCGACTGCGCGCACTCAACATTGTTGTACTGCACGTACCAATGAACCGTGAAAGTACAGTCCTGGGTCACCCCAACGCTTGTAAGGAAACCGACCACGGATCGAGTTCTTGATAATACTGTGTTCTAGAGGTGTATCTTCAAAGAAAGACCATCCAATAGGGAAAGCTTGCTTATGACTCAGTACTCCGCGGGATCTGGACCCTACATGGATGTGACTTCCTAGCGCGTGTCCACCGGCGGGTTAGAGCATTAGAAAAAACTCGTGCGGGCGATTTGCAGTCACATCTGCTTGGTTATTCGGCGCAGTTTACTCACGGTTCTGCGGATAGTCGGTTGCCATCTATCGTCATCTCCAAGGCCGATTGCGGCATTCGTCCCATTTGGGCGTTGATGGAGACCCATCGCATGGCTGCAGCTTAAAAACAACGGAGGATGATGCCGTAGGCTGTAGAATGCAAGCGCGAGCGATCGTGATCGCTACCGATCCAGTCTTCCATAGTTGGATTCTGCTAGTAGTAGCGTAGCGGATAATATGCGGCACGTGCAGCGTGGGCGTGAAAATCATCGAGGATGTGTGGATCTAGTTGGGACGTTGACTCCAGCTTATGAGAGGAATATTAGTAGCAATAAAGCGCGCGCGGGCAACCGGAACAAGGCCTGCCGTGGTCGTATCGAGTCCAGGGGGTACGGACTGAGTGCGGTTAAGAACTGTCGTCTTAACCACGTATCTCCTCCTAGATCCTATTTTGTAAATTAGTGATTGCGCTACGGACCCTTACAACCTGAAAGAGGTGTTAGGGATATGAGATAATCGAACTGATCTCGGGCGTCCCGCTAGCCCGGTTTCGTACCGGGTTTGTTGACTTATGTCCATTGTACTTCAACGGTATCCCACTACGACGTAGCGACATCTGTTTGGTATGGCATCACTTGATCCAGCTGGTTCTGGGTATGCTCGACTCCGAAATGTTGACTGGGAATGAATGGTTTAGTAGGTTTGTCTGCGAACCCAGATGGCTTGCAAGCACTGCGCAGCCGAAGACAATGTACCGTAGACAGACTGACGCACACCAAAGATTTTGCTCACTCGTGGCTTTGAATTCGCGAGGACCGGGTAACAGCGGGACGGAGGCGACGGGCCCTGTCGACGACGAGCTGCCGACTAAGGGCTCACTTTACTTTCAGGGACTCGATAATGGTTCCCTTAAGCGTCTAAGTAGCCCGTACAGTCTGCGCCCTTGCTCCACTTGGTTTCTGGATGCCCTTTACGCGGGACGTCCAGATGCCACTCGGTGACGAAGAGTTTGGCGAATGACATACGTTGCGTTTGATTAAGGCATCGCTCGCTTGTCTCAGTGCTGAAGGACCTACCACCCTCAAGATCTCCTAATACACAAGACTCAGCGCAAAGGTCCGGTTGCTGCTCCCCTTTCACCCGGTCCCCTCGTGGCATAAGGTTGTCGACCGCCCTTGGTTTAATAATGGTCGCCACGTGAGCTTCTACCTTCCCCGAGAATGGGCTAACCCTAGCTGATACATCGCTGGGAGCGGGCTGCCGAGAAACTAAATCAGGGTTCATGGTGTCCGATTTGAAATCCGTGCTCCCTTAGCCTGCGGGAACGACCCGTAATGGCCGTTCTGGAGTTAGTTAACCAGACCAGCCGTCGCACTAATTTGCTCGCCGTTGACATTATGCAGAGAACAGAGGGCAGTACGCCAGGAATTGCGCCCGTCAACTTTTGAGCAATAATATGCCTACCTTATTATAGTCTAACAGAAGTACCTAGCGTGGTGGCAGACGGCCGATAGGCCTGTAAAGGCCATGGAGGCAGGTGTACCATCTACGGGAAGTATACGTTCGCGGAGAATACAATCTTAAATTTTCGTGGGGAATATCACATGGAACGTGTCACCATAAGCCATGGACTGTCCATGTCTATTAGGATCCgcgccgcgggccgcAACCAAAACCAAgcggcccgcggcgcGGAT

>cc6m_2709_t7_ecorv (Curlcake 4)

ATCATAATTAATACGACTCACTATAGGGAGACCGAGACTCTCGCCCCTCGAAGCGATCCACCCCGCATCCTAAACCCAGTCAAACTCCTGGACCGCGCTCCCTTGAGACTAGGCGATGCTCCCTCGCCGTGGTGTTTTCTCTAGCGGTATAGCGCTTCTGTCTTCCTATGGGCCGACTAGAATGACTGTCATCACTGGCATGGCATCAAGGGCCAACCTGCGTGGTCTGATTAGCTTGGTTAGGGGCTTGCCGATGTATTTAGCGCGCGTTTCGAAACAGCGGTTTCAACATTGGCCATAGCTACCTACGAATCCCTACGATTGATACACGGTCGGGACAAATGATCCGACCAAGGTGCCTCACTGGTGGGAAGTCTAGATTCATCCGTGGGACATCGGACAGTCCTCCGTTATGCGAACCTATGACTCCCACCCCAAAGGGCTAAACAAAGACATTCCCGTACGCGAGTAGAGGGAGCCTTAACCGCAGTGGGATGCTAGGATCGGCTAAGGGTTTCTCCTAATAGGCGGGAGTTTCTTTCAATGAATACATTGACTCCTCACCTGCGTAAGTTGCGAACCTGACGCTTGCGTGATAAATCCCAGGTTCGAACGCTAGGAGACGAGGCTGCTGCAGGCGGCTGCAGTTGGAAACCATGGATAAATTGCGCCACCGGGCTACTTAATGCCTGCGCTCCAGCCCTTTGAGTTTTTGCGCGCGATCCAGTAGAATCCTCCCCGTCGGAATTAGGCCTGTGGCGCGCGGTGCACCGAGAAATTAAACTGTGGCTCACAACGGCTAGGTTTTTCTGGAGCATAGCAGATTGCCTCATGTCCATCGGGCATACTGGTGATTGTCCCAGCGTCCTCAATTTTTTTAACGCTAAGCGAGTAATGCCATCGTGGCGAGTGAATGTCGGAGGTGGAACACTAACTAGGTTAGTGGCCGGCTCCCGGCCCAATTCGGACATGCTGTAACGTTAAGATTAGTAAGACAGCTGTCGTTCTAGCACATCGGCAAGGTTATAGTTTTTCGGTTGAATGCTCACGTCCCTAAGCGGCAGGAATAGTATAGCTTTGGGCTCTTCATGCGGAGATCACCTCATTCTGCTTCTAACTCAGGGGCCTACCACGGTACACCGCCACATGTCGAGACTTAGTCTTTTTGACGGCGGAACATCTCTCAGGCGTTCCAATCGGGCGATTAGAGCACCGAGCGTTACTTAGGATGGGTAACATTTGTATGACTAAGATCACTTGAAATAAATACAAGCGGGTACGTTCGAGACGAAACGTGTACATCAGGTCACGTCGTGCCGAAGCGTACCCTTCGTCCTGATGTCGCAACACTACAAGGTCTGCCGGAGCCTAAGTTAGTGCTAGACAAGCCATGCGCCGATAACTAAAGCAACGGTCGGTTTCCTCCAGTCACGTTCTACGTTACTCAGGATTTAGCATAGCCTAGAGACAGCAGCGAGTAGTATAATACAGCGTCGGCCGACCCCAGCTAAGGACCGTCTTCTTCGATATATGGCGCCCCAAGCTTCTGAACAGAGGGGATCTCGCTCGCCCCTATATGTTCTGGTACTTACTAAACGAAAAGACAGGGTTTTTGGAGTCAAGCCTAAACTGGTGCAATGACCCCTAGCTACTGAGGCCCTGACATTGGTTTGTAGAACTGCTCGGGTGTACCGTCCACAGGTACAGCAAATCACGTCACTTGAGAGCGGTCCCGTGCTGTGTTACGGCACAATAGGCACCTTTCGTTCAAAGCATTGAGAGACGTTCCCCGAGGCGACCGGGTCACGCAGAAGAGGTCGGTATTCGCAGCCAAAGCATAACCCGGTCCTCCTCAGAACAGCAGGACTCAAGCATAGGGACAGTGTTAGCCAGCCCAAGAAGGCAGCGGCGATTGCCGACCATCTTCCCTATAATGACAAGACCTTTCAGAGTGGACGTCTACTATGTCTTGGACTGCACGGGTGTCCTACGCATCGCGTGGGTCCAAGTCCCATTAGCAACTTCTCTCCCGTCGGTACAATGTTACAGTTAAGCAGCGCCTCTCAATCGAGAGGCTGCACGGGAGCTCGAGAGGCGGTATCGAGGGGGTAGGCCGTAAAATCGCTGACTCAAACGACTATAGGAGGCGTGTTGCATGTTTAATTAAAGCCATCGTCCAATTTTCATAGCTTAAGCTAACCCGGCCACCTTGAAGAAAAACAATTCCTTACGAATGTGGCACACTGTTCCATAGAGACAGGGGGGAGTTCCCTCCATAAATCACTGTTCGTATCCGAGGTCACTCGAATGGAGTGTACACTAAGCTTACGAGGGTGTACTGCACGCAATAAGTAACATAGCCTCCAATGTTGGATTGTGTTCTACCATGGTGTGGTATCAGTCGCTGCGCTGAGTGAGTCAAGAAATCGGGAGGTAAGTTTAGCGAAGTAAGTATTGCGCGTAGTCGACCCGGATGCCCACAATCATCCATCGACGACCCCTCTTTTACTTTATATCCTTGCCGTAGGCATGGTGAGGCCATTAGCGGGTAATCACCCTCTTAGTCCATCTCGCGCCATCGAATTTCATTATACTCCGCCTTGGAGACTTACATTAGCAGTCTACGCTCTCGTTGCGCGGTCCACTCACTACTATCTGACCACTCGCAAACATCGAACCTGCGGGTACACTGGTCAGCCAAACAGGATGCCGGACAGACAACCGAACAAGAGCATGTAGGGATCCgcgccgcgggccgcTCTTCCTCTTCCgcggcccgcggcgcGGAT
